# Potential impact of neonicotinoid use on Northern bobwhite (*Colinus virginianus*) in Texas: A historical analysis

**DOI:** 10.1371/journal.pone.0191100

**Published:** 2018-01-11

**Authors:** Hannah M. H. Ertl, Miguel A. Mora, Donald J. Brightsmith, Jorge A. Navarro-Alberto

**Affiliations:** 1 Department of Wildlife and Fisheries Sciences, Texas A&M University, College Station, Texas, United States of America; 2 Department of Veterinary Pathobiology, Texas A&M University, College Station, Texas, United States of America; 3 Departamento de Ecología Tropical Universidad Autónoma de Yucatán, Yucatán, México; Northwest Fisheries Science Center, UNITED STATES

## Abstract

The widespread use of neonicotinoid insecticides in recent years has led to increasing environmental concern, including impacts to avian populations. In Texas and across their range, Northern bobwhite (*Colinus virginianus)* habitat frequently overlaps cultivated cropland protected by neonicotinoids. To address the effects of neonicotinoid use on bobwhites in Texas, we conducted a historical analysis from 1978–2012 in Texas’ ecological regions using quail count data collected from North American Breeding Bird Survey and Texas Parks and Wildlife Department, and neonicotinoid use data from the U.S. Geological Survey. We considered bobwhite abundance, neonicotinoid use, climate, and land-use variables in our analysis. Neonicotinoid use was significantly (p<0.05) negatively associated with bobwhite abundance in the High Plains, Rolling Plains, Gulf Coast Prairies & Marshes, Edwards Plateau, and South Texas Plains ecological regions in the time periods following neonicotinoid introduction (1994–2003) or after their widespread use (2004–2012). Our analyses suggest that the use of neonicotinoid insecticides may negatively affect bobwhite populations in crop-producing regions of Texas.

## Introduction

Northern bobwhites (*Colinus virginianus*; hereafter, bobwhites) are grassland birds frequently associated with agriculture [1, 2] and are known to feed on the seeds of agricultural crops [3]. Adults are predominantly granivorous, but will consume green vegetation and invertebrates. Chicks and breeding females consume a higher percentage of invertebrates to meet the protein requirements of growth and reproduction, respectively [4].

Despite their important social and economic value, bobwhites have experienced range-wide declines for decades, and have been considered near threatened since 2004 [5]. Breeding Bird Survey (BBS) analyses indicate that Texas bobwhite populations had an overall increase of 3.3% per year from 1966–1979, and have decreased 4.7% per year from 1980–1996 and 5.8% per year from 2001–2011 [6, 7]. Habitat loss by agricultural intensification and other causes has been proposed as a primary driver of bobwhite decline [8, 9]. Other factors have also been implicated in regional bobwhite losses, including drought [10], epizootics and parasites [11], local over-harvest [12], over-grazing [1], and the advance of red imported fire ants (*Solenopsis invicta)* [13]. Over the last 20 years evidence has emerged that broad-spectrum pesticide application may contribute to grassland bird decline [14–16], and that neonicotinoids may contribute to bird population losses [17].

Neonicotinoids are a relatively new class of insecticide. They were registered for use in Texas in 1994 and became widely marketed throughout Texas and the U.S. in the mid 2000’s. There are seven neonicotinoid compounds currently on the market, all of which exhibit systemic properties that allow them to be absorbed and distributed throughout a plant as it grows, making the plant toxic to insects and protecting it throughout the growing season [18]. Neonicotinoids act as agonists against postsynaptic nicotinic acetylcholine receptors in the central nervous system, and variation in the functional structure of vertebrate and insect nicotinic acetylcholine receptors facilitates their selective action towards insects [19]. Their popularity as the most widely used class of insecticide in the world is partially attributable to this selective action, which results in a lower vertebrate toxicity than their predecessors (e.g., organophosphates and carbamates). Neonicotinoids are registered for use on cereals, fruits, ornamentals, vegetables, cotton, vines, potatoes, and for home, lawn, and veterinary purposes. They also have applications in biological vector control [18] and are frequently formulated with mixtures of other pesticides (e.g., fungicides), especially when applied as a seed treatment [20].

Neonicotinoids are used in a variety of applications (e.g., foliar spray, soil drench, trunk injection, etc.), but are primarily used as a seed treatment. Since their introduction in the mid 1990’s, the prophylactic application of insecticidal seeds treatments has increased exponentially. In 2008, neonicotinoids comprised 80% of the insecticidal seed treatment market [21], and virtually all neonicotinoid use on corn, soybeans, and wheat in the U.S. is now applied as a seed treatment [22]. When applied as a seed treatment, only ~5% of the active ingredient reaches the target crop, while the other ~95% is lost to the environment [23]. As neonicotinoids are highly water soluble (log K_ow_ -0.55 to 1.26) [24] and have long half-lives (up to 545 days in soil and 40 days in water) [25, 26], seed treatments facilitate their entrance, transport, and persistence in the environment. At least twenty-nine independent studies in nine countries across the world have identified neonicotinoids in surface waters, including detections made outside of the growing season and outside of cultivated croplands [27].

Neonicotinoids were initially regarded for their high insect specificity and low vertebrate toxicity, but concerns have emerged in recent years regarding their effects on pollinators [28–32], other non-target organisms [17, 33–37], and ecosystem functioning [38–40]. These concerns sparked a review and 2-year moratorium on imidacloprid, clothianidin, and thiamethoxam in the European Union [41], and prompted the U.S. Environmental Protection Agency to review the impacts neonicotinoids have on pollinators in the U.S. [42].

The effects of neonicotinoids on avifauna are of particular interest and concern in the present study. Laboratory analyses indicate that birds exposed to various neonicotinoid compounds at field-realistic levels (i.e., dosage consistent with the manufacturer’s suggested application rate) elicit signs of oxidative stress, immunotoxicity, degenerative changes in the liver, disruption of the pituitary-thyroid axis, and alterations in reproductive ability including fewer and fragmented germ cells, reduced fertilization, eggshell thinning, delayed embryonic development and egg laying, severely reduced clutch size, and immunosuppression in adults and offspring [43–47]. Furthermore, neonicotinoids may cause prey-based collapses, as illustrated by studies of neonicotinoids and other insecticides [14, 35, 48]. Recent investigations have suggested that bobwhite and scaled quail (*Callipepla squamata*) are exposed to neonicotinoids in the Rolling Plains of Texas and Oklahoma [49], and cases of wild bird poisoning and mortality have resulted from the ingestion of neonicotinoid-treated seeds and contaminated grubs [17, 50–52].

The widespread and frequent use of neonicotinoid insecticides in bobwhite habitats warrants a thorough analysis of the relationship between bobwhite abundance and neonicotinoid use in the state of Texas. Therefore, our objective was to analyze long-term data in each of the ecological regions of Texas to characterize the relationship between bobwhite abundance and neonicotinoid use in Texas. We hypothesized that bobwhite abundance would be inversely related to neonicotinoid use in regions where neonicotinoids are heavily applied, but that no relationship would exist in regions of little or no neonicotinoid use.

## Methods

**Fig 1:**
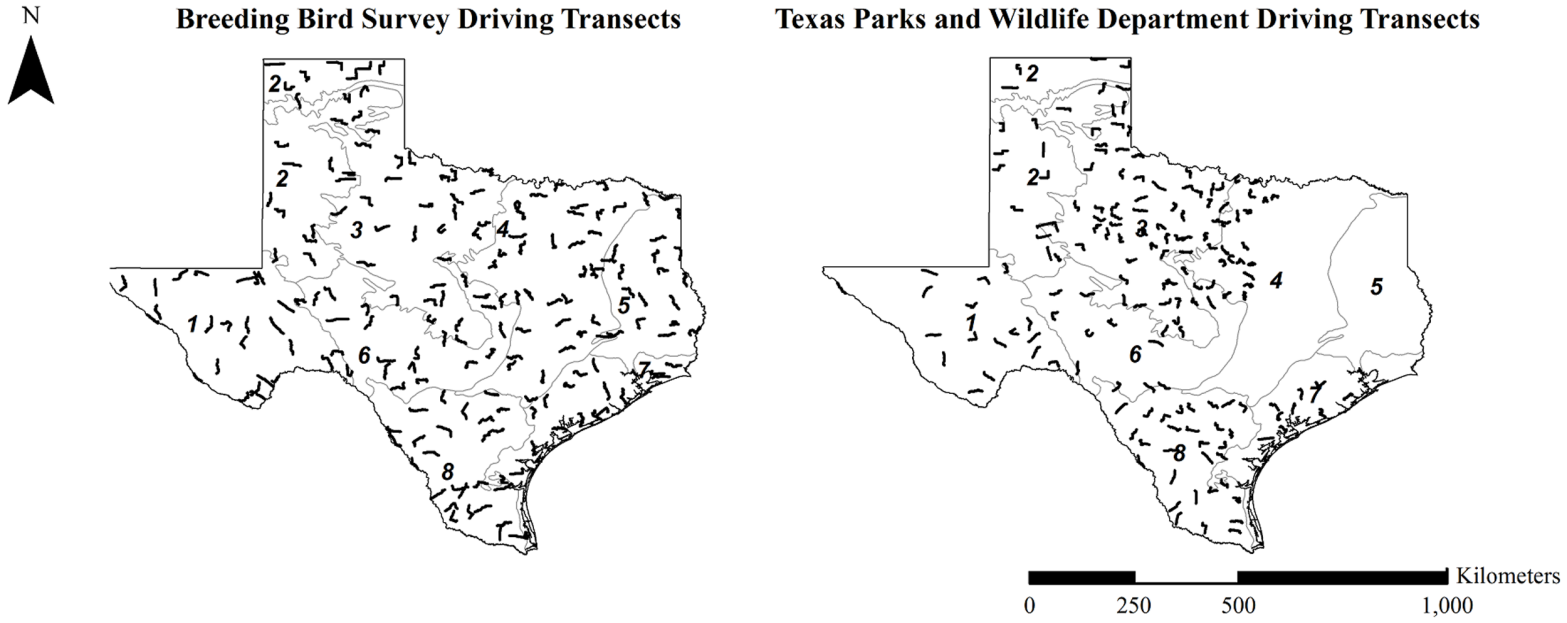
Distribution of Breeding Bird Survey and Texas Parks and Wildlife Department driving transects within Texas ecoregions. 1) Trans Pecos, 2) High Plains, 3) Rolling Plains, 4) Cross Timbers & Prairies, 5) Piney Woods, 6) Edwards Plateau, 7) Gulf Coast Prairies & Marshes, 8) South Texas Plains.

To determine the potential effects of neonicotinoid use on Texas bobwhites, we utilized available data on bobwhite abundance, neonicotinoid use, temperature, precipitation, and land use in a statistical analysis for the years 1978–2012. This analysis is limited by quail abundance data, which was not available before 1978 and neonicotinoid use data, which was not available after 2012, at the time of our study. Our study areas included each of the ecological regions (hereafter, ecoregions) of Texas excluding the Trans-Pecos, which is the western periphery of the bobwhite range (Fig 1). We combined the Cross Timbers, Post Oak Savannah, and Blackland Prairies into a single ecoregion, “Cross Timbers & Prairies,” to align with data reporting of environmental variables.

### Construction of study plots

Texas Parks and Wildlife Department (TPWD) and Breeding Bird Survey each record quail counts during annual surveys conducted along driving transects. We used these driving transects to develop study plots from which we gathered spatial data for our analysis. Driving transects were obtained online [53] or directly from Texas Parks and Wildlife Department (pers. comm., M. Frisbie, TPWD, 2015). They were imported into ArcGIS 10.2.1 [54], and re-projected into NAD 1983 UTM Zone 14 N. Plots were constructed by placing a 0.5 km buffer around driving transects, and a total of 165 BBS and 143 TPWD plots were included in the analysis. Breeding Bird Survey plots averaged 41.0 km ± 2.8 km in length with a low of 30.2 km and a high of 49.1 km, and Texas Parks and Wildlife Department plots averaged 32.5 km ± 0.38 km in length with a low of 30.9 km and a high of 33.0 km. The Breeding Bird Survey and Texas Parks and Wildlife Department were unable to consistently survey all transects over the years; therefore, when a transect was not surveyed in a given year, the corresponding plot was omitted from the analysis for that year.

### Data collection

#### Quail abundance

We obtained quail abundance data from the U.S. Geological Survey (USGS) Patuxent Wildlife Research Center online database [55] and directly from TPWD (pers. comm., M. Frisbie, TPWD, 2015). Survey protocols varied between organizations. Breeding Bird Survey volunteers conduct general avian surveys in June by stopping 50 times for exactly 3 minutes at equal intervals along driving transects and recording visual and auditory observations of all birds [55]. Texas Parks and Wildlife Department biologists conduct quail surveys in August by driving at 32.2 km per hour along driving transects and recording visual observations of quail [56]. Because TPWD study plots were poorly distributed across the Edwards Plateau and Cross Timbers & Prairies ecoregions, and omitted the Pineywoods ecoregion altogether, we report only the BBS analysis for these regions.

#### Neonicotinoid use estimates

To evaluate neonicotinoid levels in each plot we obtained USGS ePest High values of estimated county-level neonicotinoid use [57, 58] for all neonicotinoid compounds applied in Texas. The USGS calculates ePest High values using data from USDA Crop Reporting Districts. Unlike ePest Low values, ePest High values incorporate data from neighboring districts when data for a given Crop Reporting District is missing. The summed total of all compounds was used to obtain a single value of estimated annual county-level neonicotinoid use. Total neonicotinoid use within each plot was calculated by multiplying the cumulative county neonicotinoid use by the proportion of county cropland that fell within each plot.

#### Climate

Research has shown that the Palmer Modified Drought Index (hereafter, drought index) may be used as a good predictor of quail abundance [59], while breeding season (April through August) precipitation and summer (June through August) mean maximum daily temperature are predictive of quail productivity (i.e., age ratios) [60]. To characterize the climatic conditions within each plot, we obtained the following data for each year of the study period: (1) raster images of precipitation for each month of the breeding season; (2) monthly raster images of summer mean maximum monthly temperature (daily values were not available) from the Parameter-elevation Regressions on Independent Slopes Model online databank [61]; and (3) monthly summer drought index values, obtained from the National Ocean and Atmospheric Administration [62]. Precipitation and temperature data were statistically modeled raster graphics and are the U.S. Department of Agriculture’s official spatial climate data. Drought index values range from -5.0 (severe drought conditions) to +5.0 (extreme wet conditions) and are calculated using precipitation, temperature, evapotranspiration rates, and other climatic variables [63].

Monthly precipitation and mean maximum monthly temperature rasters were imported into ArcGIS 10.2.1 [54] and re-projected into NAD 1983 UTM Zone 14 N. Zonal Statistics was used to identify mean precipitation across each plot for each month of the breeding season. These values were then summed, yielding total breeding precipitation. Summer mean maximum monthly temperature was calculated by averaging the maximum temperature in each plot for each of the summer months using Zonal Statistics. Drought index values are available regionally in areas closely resembling Gould’s ecoregions [64] (S1 Fig). Drought index values were averaged over summer months for each ecoregion, resulting in a single value representing the summer drought index.

#### Land use

As habitat fragmentation by agricultural intensification and urbanization is frequently cited as a major contributor to quail decline, we used total cultivated cropland and total developed area in our analysis. To identify these land use variables in our plots, we used statistically modeled land cover raster images obtained from the USGS Earth Resources Observation Systems lab [65]. Land use rasters were imported into ArcGIS 10.2.1 [54] and re-projected into NAD 1983 UTM Zone 14 N. We reclassified land use into two separate binary raster images for each year of the study period: (1) cultivated cropland—non-cultivated cropland and (2) developed—undeveloped. Tabulate Area was used to calculate the total cultivated cropland and total developed area falling within each plot.

#### Supporting shapefiles

Supporting boundary layers including state, ecoregion, and county boundaries were obtained online from Texas Natural Resources Information Systems [66]. These vector files were imported into ArcGIS 10.2.1 [54] and re-projected into NAD 1983 UTM Zone 14 N prior to their use in any operations.

### Statistical analysis

**Fig 2:**
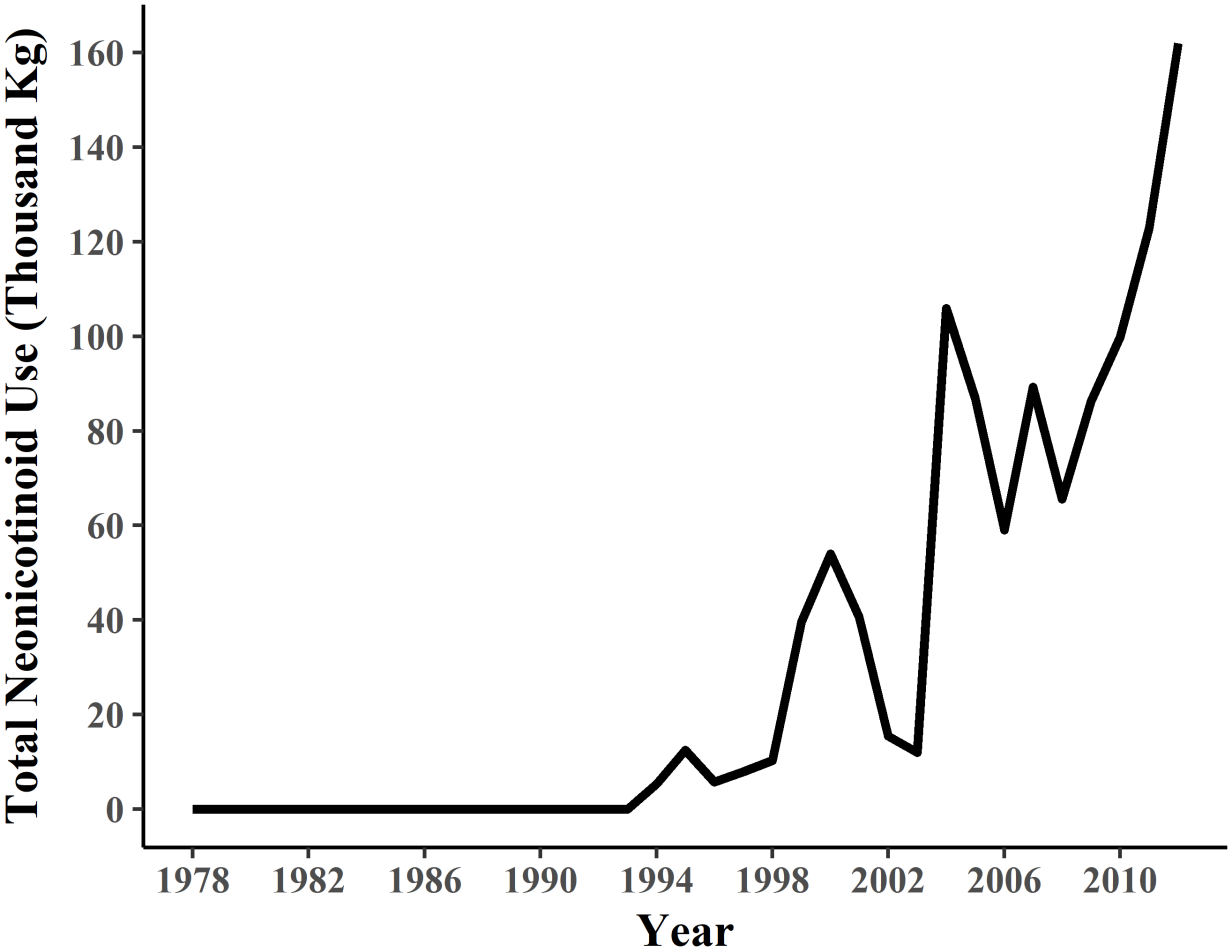
Temporal trend in neonicotinoid use in Texas. USGS ePest High estimates for total neonicotinoid use in the state of Texas from 1978–2012. Statistical analysis was split into three time periods based on overall levels of neonicotinoid use: prior to neonicotinoid introduction (Pre), directly following neonicotinoid introduction (Light), and after the widespread use of neonicotinoids (Heavy).

Because survey protocols varied drastically between BBS and TPWD (e.g., driving transect lengths and observation procedures), and could influence model outcome, datasets from both organizations were analyzed separately. Analyses were divided into three time periods according to overall neonicotinoid use patterns (Fig 2): prior to neonicotinoid introduction (1978–1993), directly following introduction (1994–2003), and after their widespread use (2004–2012). These are respectively termed BBS-Pre/TPWD-Pre, BBS-Light/TPWD-Light, or BBS-Heavy/TPWD-Heavy. Variables included in the statistical analysis are detailed in Table 1. Computational analyses were conducted using R Statistical Programming Language, version 3.2.3 [67].

**Table 1:** Description of variables used in historical analysis.

Variable	Category	Description	Source
Bobwhite abundance	Bobwhite (Dependent)	Bobwhite count in study plot (number of individuals of any age).	M. Frisbee, TPWD, 2015; [55]
Summer drought index	Climate (Independent)	Summer Palmer Modified Drought Index within study plot.	[62]
Breeding season precipitation	Climate (Independent)	Sum of breeding season precipitation within study plot (mm).	[61]
Summer mean maximum monthly temperature	Climate (Independent)	Mean of summer maximum monthly temperature within study plot (°C).	[61]
Total cultivated cropland	Land Use (Independent)	Total cultivated cropland within study plot (km^2^).	[65]
Total developed area	Land Use (Independent)	Total developed area within study plot (km^2^).	[65]
Total neonicotinoid use	Pesticide (Independent)	Sum of neonicotinoid application within study plot (kg; ePest High estimate).	[57,58]

To identify distribution patterns in the data, we constructed histograms and q-q plots of all variables. It was apparent that quail abundance was zero-inflated; thus, to describe this response as a function of the explanatory variables (Table 1), we used zero-inflated generalized linear models [68]. A log-link was used in all models requiring a link function.

In most cases, six different models were generated to describe trends in each of the ecoregion-level analyses: one model explaining each dataset (BBS and TPWD) for each of the three time periods. We excluded TPWD data from the analysis in the Cross Timbers & Prairies, Edwards Plateau, and Pineywoods ecoregions due to insufficient data or poor geographical distribution of driving transects.

The model selection process consisted of three steps: 1) The observed response was fitted to a generalized linear model containing the six quantitative effects shown in Table 1, assuming a negative binomial distribution for the random error, and used stepwise regression in both forward and backward directions to identify the combination of variables that yielded the lowest AICc (Akaike information criterion corrected for finite sample sizes) value [69]; 2) The set of predictors selected in step 1 were included in generalized linear, zero-inflated, hurdle and generalized additive models, all of them with negative binomial distribution for the error; and, 3) AICc weights (quantifying the weight of evidence in favor of a given a model) were calculated for all candidate models to select the model that provided the largest AICc weight.

Given the diversity of sampling locations and data sources for all the bobwhite data set, it was practically impossible to get the same set of predictors for the selected models. Therefore, to better summarize the influence of each predictor on bobwhite abundance, we enumerated the total number of models fitted to predict quail abundance, and the number of times each predictor’s coefficient was positively or negatively associated to the response.

## Results

Of the six predictor variables tested in this study, the strongest negative association was between bobwhite abundance and neonicotinoid use (Tables 2 and 3). Total developed area and total cultivated cropland were also negatively associated with bobwhite abundance, although to a lesser extent than with neonicotinoid use. In contrast, summer drought index and summer mean maximum monthly temperature were positively associated, while breeding season precipitation did not show a significant positive or negative association with bobwhite abundance (Tables 2 and 3).

**Table 2:** Overall influence of predictor variables on quail abundance across all best-fit statistical models. Percent of models (out of 32) positively or negatively associated with quail abundance.

Variable	Positive association	Negative association
Total neonicotinoid use	5%	62%
Total developed area	19%	38%
Total cultivated cropland	22%	31%
Breeding season precipitation	16%	16%
Summer mean maximum monthly temperature	44%	16%
Summer drought index	47%	9%

**Table 3:** Coefficients of best-fit statistical models.

	Summer drought index	Breeding season precipitation	Summer mean max. monthly temperature	Total cultivated cropland	Total developed area	Total neonicotinoid use	Model Type
**High Plains**							
BBS-Pre				-0.021*	0.372***		Hurdle
TPWD-Pre	0.105	0.007**	0.424*	-0.001	1.06		Zero-inflated
BBS-Light			0.38***				Zero-inflated
TPWD-Light			0.014	-0.065***			Zero-inflated
BBS-Heavy						-0.032***	Hurdle
TPWD-Heavy	0.390***		0.466***			-0.069***	GLM
**Rolling Plains**							
BBS-Pre		0.002**	0.142***	-0.017***	-0189		GLM
TPWD-Pre	0.136***		0.061	-0.01	-0.84***		Zero-inflated
BBS-Light	0.135**		0.299***	0.009	-0.212**	-0.615***	GLM
TPWD-Light	0.122**		0.008			-0.006	Zero-inflated
BBS-Heavy	0.117**			0.036***	0.161**	-0.058***	GLM
TPWD-Heavy	0.207***	-0.004***		0.021	0.03	-0.056*	Hurdle
**Cross Timbers & Prairies**							
BBS-Pre	-0.052*			-0.029***	-0.03*		GLM
BBS-Light	0.127	0.002**	-0.141	-0.066***			GLM
BBS-Heavy	-0.196*		-0.292**	-0.111***	-0.067		GLM
**Pineywoods**							
BBS-Pre	-0.122**			0.053***	-0.236***		GLM
BBS-Light	0.192*	0.003**	-0.232*	0.079***	-0.352**		GLM
**Edwards Plateau**							
BBS-Pre		0.003**	0.125	0.101***	-0.128		Zero-inflated
BBS-Light					-0.213***	-0.712**	Hurdle
BBS-Heavy			-0.207***			-0.038*	Zero-inflated
**Gulf Coast Prairies & Marshes**							
BBS-Pre					0.175***		GLM
TPWD-Pre	0.143**		0.251**		-2.41		GLM
BBS-Light		-0.001*	0.092		-0.088*	-0.07**	GLM
TPWD-Light		-0.001		-0.028		0.074	Zero-inflated
BBS-Heavy	0.118*		0.171*			-0.012*	GLM
TPWD-Heavy	0.194		0.297*		0.355		GLM
**South Texas Plains**							
BBS-Pre				-0.039***			GLM
TPWD-Pre	0.173***						Hurdle
BBS-Light			-0.089			-0.013***	Hurdle
TPWD-Light		-0.001		0.034**		-0.009	Zero-inflated
BBS-Heavy	0.079*	-0.001			-0.049	-0.008	Hurdle
TPWD-Heavy	0.152		0.291**				GLM

*p< 0.05;

**p< 0.01;

***p< 0.001.

Coefficients given for hurdle and zero-inflated models are count model coefficients.

Prior to neonicotinoid introduction (1978–1993), summer drought index, breeding season precipitation, and summer mean maximum monthly temperature were positively and significantly associated (p < 0.05) with quail abundance in five of the seven ecoregions tested (all but the Cross Timbers & Prairies and Piney Woods; Table 3). However, total cultivated cropland and total developed area were significantly and negatively associated (p < 0.05) with quail abundance at most of the seven regions, except for the Piney Woods and Edwards Plateau (for cropland) and the High Plains and Gulf Coast Prairie (for developed area, Table 3).

During the period of light neonicotinoid use (1994–2003), summer drought index and breeding season precipitation were positively and significantly associated (p < 0.05) with quail abundance at the Rolling Plains, Piney Woods, and Cross Timbers & Prairies regions. Summer mean maximum monthly temperature was also significantly and positively associated (p < 0.05) with quail abundance at the High Plains and Rolling Plains regions, but it was negatively and significantly associated (p < 0.05) with abundance at the Cross Timbers & Prairies and Piney Woods regions (Table 3). Interestingly, total developed area and total neonicotinoid use were negatively and significantly associated (p < 0.05) with quail abundance at all regions from which we had sufficient data for comparisons, including the Rolling Plains, Piney Woods, Gulf Coast Prairies, Edwards Plateau, and South Texas Plains. Total cultivated cropland was also negatively and significantly associated with quail abundance at the High Plains and Cross Timbers & Prairies region (Table 3).

During the period of heavy neonicotinoid use (2004–2012), summer drought index and summer mean maximum monthly temperature continued to have a positive significant relationship (p < 0.05) with quail abundance at the High Plains, Rolling Plains, Gulf Coast Prairie, and South Texas Plains (Table 3). In the Cross Timbers & Prairies and Edwards Plateau regions, quail abundance was negatively and significantly associated (p < 0.05) with summer drought index, summer mean maximum monthly temperature, and total cultivated cropland. Total neonicotinoid use and quail abundance were significantly negatively associated (p < 0.05) in all regions. Overall, ten of the 14 statistical comparisons between neonicotinoid use and quail abundance for the period 1994–2012, indicated a significant negative association (p < 0.05) at most of the Texas regions from which data were available. In three of the four cases where the results were not significant, the coefficients were still negative (Table 3).

## Discussion

In all instances where neonicotinoid use was significantly associated with bobwhite abundance, it exhibited a negative influence on bobwhites. All other variables in the historical analysis exhibited both positive and negative associations with bobwhite abundance across the best-fit models, indicating spatial and temporal differences in the way variables influence bobwhite abundance.

Unsurprisingly, at least one climate variable was included in over 80% of the best-fit models, indicating a strong influence of climate on bobwhite abundance. In accordance with previous research [59,60] summer drought index tended to positively influence bobwhite abundance. Moist conditions often produce improved habitat quality and an increase in usable space, resulting in irruptive or at least improved quail production [70]; however, under these conditions bobwhites compete with irruptive populations of other animals (e.g., rodents) for resources, and in extremely wet conditions flooding can destroy nests and cause birds to drown. An assessment of bobwhites conducted in the Gulf Coast Prairies & Marshes in 2015 (a record rainfall year in Texas) identified drowned, radio-collared hens and flood-destroyed nests (per. comm., N. Silvy, Texas A&M University, 2016). This disparity in too little or too much rainfall may explain why breeding season precipitation was negatively correlated with bobwhite abundance in 5 of the 10 best-fit models in which it was included.

We were surprised to find that summer mean maximum monthly temperature was positively associated with bobwhite abundance in nearly half of the best-fit models. Bobwhites’ body temperature is naturally precariously close to their upper lethal limit [71]. It is therefore critical for them to avoid heat stress during the summer months, and past research has identified a negative relationship between summer mean maximum daily temperature and bobwhite age ratios in South Texas [60]. In our analysis, summer mean maximum monthly temperature averaged 34.5°C ± 1.7° (94.1° F ± 3.1°) and showed a slight (< 1°C) increase over time periods. In the High Plains, Rolling Plains, Gulf Coast Prairies & Marshes, and South Texas Plains all significant (p<0.05) correlations between abundance and temperature were positive, while significant (p<0.05) correlations in the Cross Timbers & Prairies, Pineywoods, and Edwards Plateau were all negative. The disparity in our results and those of others warrants further investigation into the effects of summertime temperature on bobwhites, with careful consideration of regional differences.

Since habitat fragmentation by agricultural intensification and urbanization is well established as a major contributor to quail decline [9], we expected both land use variables to elicit a negative effect on bobwhite abundance. Both total developed area and total cultivated cropland were more often negatively associated with bobwhite abundance. We suggest this may be associated with the size and structure of our study plots and the structure of developed areas and cultivated cropland. Bobwhites primarily utilize weedy fence and hedgerows in cultivated areas. Because driving transects (i.e., roads) break up cultivated fields, weedy fence and hedgerows are well represented in our 1 kilometer-wide study plots containing cultivated cropland. The high proportion of fencerows in our study plots in comparison to the vast majority cultivated cropland (where weedy fencerows are becoming increasingly sparse), may positively bias the number of quail counted in a survey. Conversely, developed areas, although not uniform in structure, typically do not contain boundaries along roads that would bias the number of quail seen along a random transect in a developed area.

Total neonicotinoid use exhibited a negative influence on bobwhite abundance in over 60% of all models included in the time periods after their introduction. None of the best-fit models indicated a significant (p<0.05) positive association between neonicotinoid use and bobwhite abundance in any of the time periods. In areas where neonicotinoids may contribute to bobwhite decline, we would expect to see a statistically significant inverse relationship between these two variables during the time period after the widespread use of neonicotinoids (2004–2012), and possibly the time period directly following their introduction (1994–2003). The High Plains, Rolling Plains, Gulf Coast Prairies & Marshes, South Texas Plains, and Edwards Plateau all exhibited a negative relationship between bobwhite abundance and neonicotinoid use during at least one of these two time periods.

All of the ecoregions mentioned above produce crops (e.g., winter wheat, upland cotton, corn, sorghum, sunflower, and soybeans) that are utilized by bobwhites [72]. Bobwhites are known to consume and sometimes prefer the seeds of farm crops [3], and forage from field margins bordering cultivated cropland [8–9, 73–74]. In 2014, Texas growers harvested 2.2 million acres of corn and 2.3 million acres of sorghum from the High Plains, South Texas Plains, Gulf Coast Prairies & Marshes, Cross Timbers & Prairies, and Edwards Plateau. In the same year, 2.2 million acres of winter wheat and 4.6 million acres of cotton were harvested from these regions as well as the Rolling Plains, 92 thousand acres of sunflower were harvested mainly from the South Texas Plains, and 140 thousand acres of soybeans were harvested from the Gulf Coast Prairies & Marshes [72, 75]. Each one of these crops is protected by the neonicotinoid class of insecticide, and in many cases neonicotinoids are the most commonly applied insecticide used to protect these crops.

Of all neonicotinoid applications, treated seeds probably present the biggest hazard to bobwhites and other granivorous species because they likely deliver higher concentrations of active ingredient than other sources (i.e., contaminated insects or vegetation) [36]. Neonicotinoid seed treatment is a common practice for many crops planted in Texas, and bobwhites may be exposed to treated seeds not properly stored, shallowly sown, or spilled during planting. Bobwhites’ susceptibility to neonicotinoid compounds is well established [76] and onset of severe incapacitation resulting from exposure to imidacloprid, for example, is seen in bobwhites at levels between 30–60% of the LD_50_, and neurotoxic effects are usually exhibited within minutes of ingestion [77].

Bobwhites’ susceptibility to neonicotinoid-treated seeds may help explain the negative correlation we found between bobwhite abundance and total neonicotinoid use in ecoregions rich in crop production. First, the neurotoxic effects of neonicotinoids may increase bobwhites’ susceptibility to predation [78], as seen in studies of other acetylcholine-inhibiting insecticides [79, 80]. Second, their adverse effects on reproduction [43–47] could directly limit the number of offspring produced or predispose hens to clutch abandonment or reduced chances of re-nesting [81], limiting their ability to recruit a sufficient number of individuals each year to maintain populations. Many Texas crops are planted in the spring (e.g., corn, sorghum, soybeans, sunflower, cotton), and neonicotinoid application often coincides with the development of sex organs as bobwhites physiologically prepare for the breeding season. Neonicotinoids are also persistent in the environment [33], and have been detected in field margin plants [82, 83] and outside of the growing season [84], potentially making them available to bobwhites throughout the year. Third, immune suppression, a common side effect of neonicotinoid exposure [43, 45–46, 85] could increase bobwhites’ susceptibility to epizootic and parasitic infestation [86]. Finally, neonicotinoid use may limit prey abundance during critical periods (i.e., breeding, brood-rearing, and over-wintering), which has previously been linked to declines in farmland birds [14, 35, 48, 87].

Turaga et al. (2015) recently analyzed 98 bobwhite and scaled quail in the Rolling Plains and determined that they are not directly affected by the use of neonicotinoids based on two lines of evidence: a lack of treated seeds in their crops and low concentrations (≤ 62.29 ng/g) of neonicotinoids in their livers. Since neonicotinoids are highly water soluble, it is likely that only low concentrations of neonicotinoid compounds will be found in organ tissues. Additionally, the authors suggest that quail may circumvent neonicotinoid poisoning due to repellent effects of treated seeds, avoidance of treated seeds, and seed husking. However, EPA scientists suggest that neonicotinoids do not elicit any initial repellent effects [88], and avoidance of treated seeds is unlikely in field-realistic conditions [17, 88] unless an animal has already experienced post-ingestion distress from eating treated seeds [89]. Also, analysis of crop contents has suggested that bobwhites do not husk seeds [90]. Like other birds [17, 50–52], bobwhites are likely to consume treated seeds, at least initially, potentially subjecting them to lethal or otherwise harmful doses of neonicotinoids.

## Conclusions

Bobwhites have undergone population declines long before the introduction of neonicotinoids; however, long-term monitoring efforts reveal that they are declining faster now than they were in the past throughout most southeastern and Midwestern states. The results of our analyses suggest that neonicotinoid use may contribute to bobwhite decline in Texas ecoregions that produce crops utilized by bobwhites. These results also could be applied to other regions of the southeastern and Midwestern United States where bobwhites are likely to feed within or near agricultural environments. It is possible that neonicotinoids have partially contributed to bobwhite declines in various regions of the U.S.

## Supporting information

S1 Fig Gould’s ecological regions of Texas and Palmer Modified Drought Index reporting regions. (TIF)
