# Supplementary figures and images for: Potential impact of neonicotinoid use on Northern bobwhite (Colinus virginianus) in Texas: A historical analysis
Source: PLoS One. 2018 Jan 11;13(1):e0191100. doi: 10.1371/journal.pone.0191100 (PMC5764362; doi:10.1371/journal.pone.0191100)

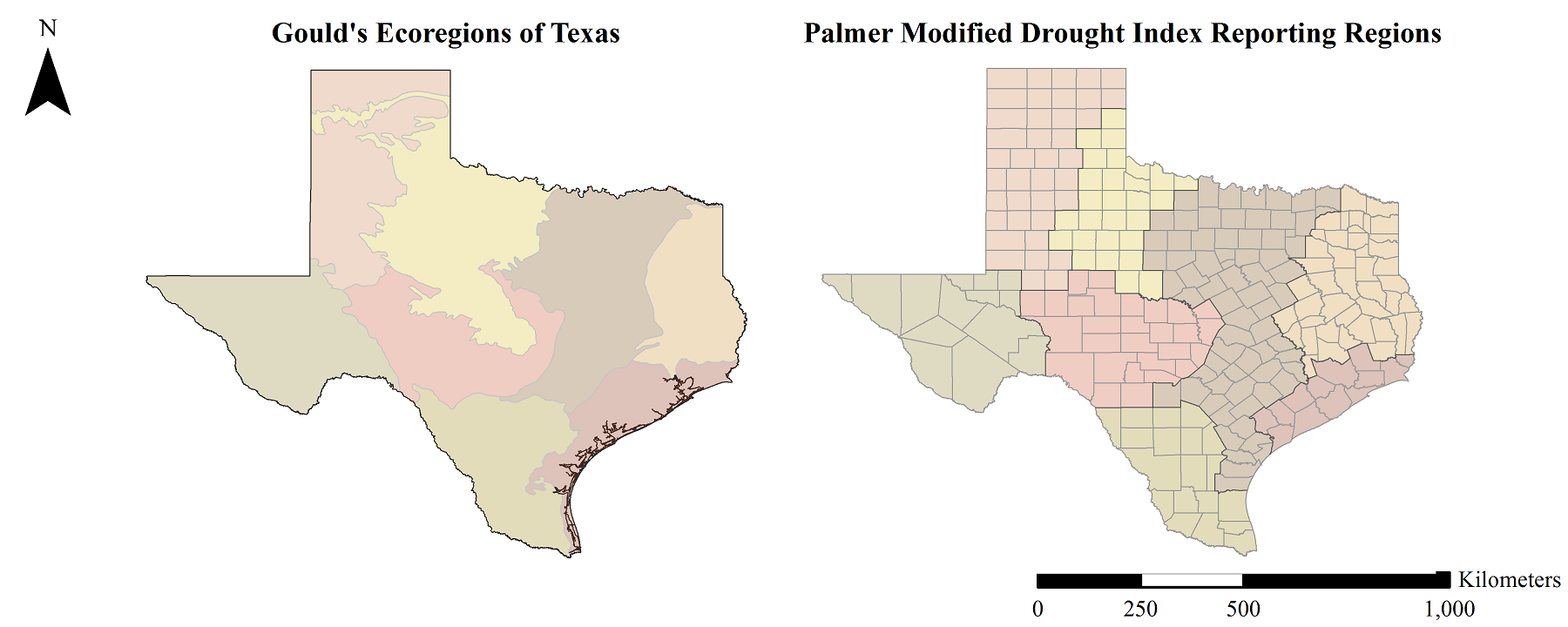

Supplement: S1 Fig — (TIF) [file pone.0191100.s001.tif]
